# Young Italian NEETs (Not in Employment, Education, or Training) and the Influence of Their Family Background

**DOI:** 10.5964/ejop.v11i2.901

**Published:** 2015-05-29

**Authors:** Sara Alfieri, Emiliano Sironi, Elena Marta, Alessandro Rosina, Daniela Marzana

**Affiliations:** a Department of Psychology, Università Cattolica del Sacro Cuore, Milan, Italy; b Department of Statistical Sciences, Università Cattolica del Sacro Cuore, Milan, Italy; Aalborg University, Aalborg, Denmark

**Keywords:** NEET, family relations, young people, social exclusion, transition to adulthood, family background, unemployment

## Abstract

This work investigates the relationship between family variables (parents’ educational level, relationship quality, intrusiveness, support, and autonomy) and young Italians’ status as NEETs (Not in Employment, Education, or Training). We used data from a representative sample of 9,087 young Italians. Each participant filled out an anonymous online questionnaire that contained several scales to measure the variables mentioned above. The results reveal that parents’ educational level and support have a protective effect on the risk of becoming a NEET for both genders. Autonomy has a specific negative impact for males while intrusiveness has a positive impact mainly for females.

While in archaic societies most transitions took place by discrete “leaps” and were accompanied by actual “rites of passage” that signaled a collective wish to mark the passage to a new condition, in contemporary modern society transitions are represented increasingly as being individual, relatively undefined (with respect to both modalities and timing), negotiable, little ritualized, and as having wide margins of choice.

The slowing down of the transition to adulthood has given the family of origin more value and power to influence during a period of the life-cycle in which, in past decades, it has tended to play only a minor role in many western societies (Scabini, Marta, & Lanz, 2006).

Young Italians, in particular, leave home at a later age on average than young people in other developed countries. The peculiarities of the Italian context can be explained from both cultural and structural standpoints. On the one hand, the presence of strong intergenerational ties is coherent with longer stays in the family of origin (Dalla Zuanna & Micheli, 2004). On the other hand, the unfavorable labor market and a welfare system that is not generous to young generations tend to discourage individual autonomy (Rosina, Micheli, & Mazzucco, 2007).

This is even true today during this exceptional social-historical moment in which the transition to the world of work has become difficult due to the recent economic crisis: “The current crisis has severely affected employment. This is of special concern to young people, who are more vulnerable to the effects of unstable economic cycles in the labour market” (European Commission, 2012).

The educational route is not free of risk either. Alongside the growing number of universities present throughout Italy offering a wide variety of programs of study and easier access even for youth from less affluent backgrounds, the probability that they will not succeed in completing their degree remains high (Bratti, Checchi, & de Blasio, 2008). According to the Eurostat (2009) report “Youth in Europe”, only about 10% of students in Italy who have a parent with a low educational level succeed in obtaining a university degree, in contrast to the European average which is more than double this value.

In this connection, an emerging phenomenon in industrialized societies having to do with the transition to adulthood is that of NEETs, an acronym for *Not in Employment, Education, or Training*: in short, young people who are unemployed or are outside the system of education and training. The NEET label generally applies to young people aged between 16 and 24 years (The Prince’s Trust, 2007), although this can vary (Rose, Daiches, & Potier, 2012). For example, Pemberton (2008b) defines as NEETs young people in the UK aged between 16 and 18 years while Chen (2011) includes those aged between 15 and 34 years in Japan and between 15 and 24 in Taiwan. This can depend on many factors: the meaning that different cultures ascribe to “youth,” the obligation to attend school until the age of 14, 16, or 18, depending on the country, as well as the possibility of easily accessing educational opportunities.

Independently of these differences, all definitions of NEET concur in identifying a category particularly at risk for social exclusion from mainstream adult life.

The results obtained by the recent Survey of Adult Skills - a product of the OECD Programme for the International Assessment of Adult Competencies (PIAAC) - showed that NEETs are the social group most at risk for depletions of key skills that are important in enabling adults to fully integrate and participate in the labor market, education and training, and social and civic life (Di Francesco, 2013; OECD 2013).

Apart from patchy employment prospects, subsequent consequences may include difficult relationships, lack of social and political participation, poor physical and mental health, drug abuse, and criminality (Atkinson & Hills, 1997; Robins & Rutter, 1990), as well as important repercussions on the wider social context (Lee, 2004; Maguire & Rennison, 2005). For those young people who have economic support from their parents, NEET status is not so much an economic problem as one of idleness, which makes it more difficult for NEETs to adjust to adulthood (Chen, 2011).

Italy, in particular, besides being the country where the phenomenon of emerging adulthood appeared first and on the largest scale, is also the European country with the third highest percentage of NEETs aged between 18 and 24 years (27.0% in 2012), after Turkey (35.0%) and Greece (28.4%) (Eurostat, 2012).

To date no studies appear to have investigated this phenomenon in depth in Italy despite its importance here. The aim of this study is to offer a contribution to fill this gap and to investigate which characteristics of family relations distinguish Italian NEETs.

## The Peculiarities of Italian Families

The slowing down of the transition to adulthood and, in particular, remaining for a longer time in the family of origin are possible only because adolescents and young adults can count on their families. We are now witnessing an essentially peaceful transformation of family relations in which, as research shows at least in Italy, families with young adults are characterized by good communication and high levels of support and relationship quality. The results of research into enmeshment and the acquisition of autonomy are more complex. In this connection, a recent study carried out by Cigoli, Margola, and Molgora (2010) demonstrated that the acquisition of an adult identity status - and, therefore, of autonomy - is determined by a) family life style, b) the young person’s status as a worker or student, and c) residence in a specific geographical location. In particular, workers - whether high school or college graduates - turn out to be more capable of managing space for autonomy with respect to family relations while students appear to experience a conflict between expectation and fear.

Manzi, Vignoles, Regalia, and Scabini (2006), comparing Italian and British young people, showed that, while for the former greater quantity of free time spent with their parents predicted better family functioning and greater well-being, for the British young adults higher levels of psycho-social well-being are associated with greater distance from the family of origin. In synthesis, investigating the relation between young people and family relationships within a specific context thus appears to be crucial.

## What Characterizes the Family Relationships of NEETs?

Qualitative and quantitative studies carried out in the UK on NEET young people aged 16 to 18 years have identified the following sets of factors: inequality variables; personal situations; family and environmental variables; bad experiences in the educational system; failures of the educational system. In particular, studies carried out by Bynner, Joshi, and Tsatsas (2000) and by Bynner and Parsons (2002) revealed five main sets of factors influencing NEET status. The majority of these are related to intergenerational or educational influences (family circumstances, such as social class; parents’ educational level; parental interest in children’s education and aspirations; place of residence; early educational attainment of children) and also showed some differences linked to gender.

Rennison, Maguire, Middleton, and Ashworth (2005) find that NEET status is more prevalent among young people whose families did not have access to high educational levels and, consequently, are not able to provide support and advice about the education system.

## The Present Study

The topic of NEETs’ family characteristics has never been studied in Italy. Yet, in our opinion, it is important to carry out such research in Italy, a country characterized by strong intergenerational family ties and weak public welfare for the new generations (De Rose, Racioppi, & Zanatta, 2008), in addition to being the birthplace of emerging adulthood.

The present work aims to fill this gap by investigating the relation between family relationships and NEET status.

## Methods

### Participants and Procedure

The sample consists of 9,087 individuals aged between 18 and 29 years (*M* = 23.22, *SD* = 3.44), taking under consideration the age bracket defined by Arnett (2000, 2006) as constituting *emerging adulthood*. In fact, this is a phase of the life cycle characterized by change and exploration of possible life directions. The individuals were chosen with a stratified sampling technique and are representative of young adults residing in Italy. Sampled individuals are equally distributed according to gender (49.0% male). 38.7% are students; 34.7% are workers. About 7% both work and study while 19.6% of the sample are NEETs.

For NEET status to reflect the dynamics of young people’s lives, it must be defined longitudinally and represent a minimum period of time that the young person is not engaged in education, training, or employment - as opposed to his/her being involved in one or more of these activities over the same period. For this reason, in the present work we considered as NEETs only young people who have been outside educational, work, or training spheres for at least six months (see, for example, Bynner & Parsons, 2002).

The data for our work comes from the database of the “Rapporto Giovani (Youth Project)” launched in 2011 by the Toniolo Institute of Advanced Studies with the involvement of the CARIPLO Foundation and IPSOS LTD as executive partners.

We used a mixed methodology for recruitment: CATI (Computer-assisted telephones interviewing) and CAPI (Computer-assisted personal interviewing) with in-depth CAWI (Computer Aided Web Interviewing). More details are available at http://www.istitutotoniolo.it

### Instruments

For the survey, participants filled out a questionnaire composed of demographic and background factors such as gender which also included details about family of origin. In addition, the analysis contains a set of family indices measuring quality of the relationship between the young adults and their parents that are helpful for establishing a link between family context and the young people’s transition to adulthood.

The variables used in the empirical analysis to predict the condition of NEET are listed as follows. These, together with Cronbach Alpha, are available in Table 1.

#### Parents’ education

We considered three categories: higher, secondary, and lower. Higher education means that respondents’ mother/father completed a tertiary education cycle, completing a bachelor’s, master’s, or doctoral degree. Secondary education indicates that the respondent’s parent obtained a 5-year high school diploma while lower education is a residual-reference category that includes primary education, lower secondary, or an incomplete upper secondary education.

#### Quality of relationship with parents

This was measured using the Italian “*Scala della qualità delle relazioni con i genitori*” (“*Quality of the Relationship with Parents Scale”)* (Scabini & Cigoli, 1992), composed of 12 items (e.g. “*My mother/my father understands me*”) (from 1=*Never* to 4=*Always*). This scale measures the perception of support between parents and child.

#### Intrusiveness

We used a translated and adapted version of the intrusiveness subscale of Green and Werner’s (1996) *CIFA* (*California Inventory for Family Assessment*) that measures aspects like coercive control, separation anxiety, possessiveness/jealousy, and emotional reactivity. This is a scale composed of 9 items (e.g. “*My father/my mother often thinks he/she knows what I am thinking without asking me*”) (from 1=*Never* to 4=*Always*).

#### Support

This was calculated using the weighted mean of two *ad hoc* items (e.g. “*Overall, how much do you feel able to count on your father’s support*?”) on a 4-step response scale (from 1 = *Not at all* to 4 = *Very much*).

#### Autonomy

This was measured using a translated and adapted version of *Autonomy Support Scale of the Perceptions of Parents Scale* (Grolnick, Ryan, & Deci, 1991) that measures autonomy perceived by children. This is a scale composed of 8 items (e.g. “*My father/my mother lets me make my own plans for what I want to do*”) on a 4-step scale (from 1 = *Not at all* to 4 = *Very much*).

### Data analysis

We used a binary probit regression model where the dependent variable is a categorical indicator taking value 1 if the respondent has not been engaged either in education or training for a period exceeding six months and 0 if the respondent is working or studying at the time of the interview. We decided to exclude from the analysis individuals who were not working or studying but who had concluded their educational or work activities less than six months before the data collection. The reason for this choice has to do with the hypothesis that the condition of NEET applies (see, for example, Bynner & Parsons, 2002) only when a long period of inactivity is ongoing.

## Results

Descriptive statistics of the sample for the variables used in the analysis are reported in Table 1.

**Table 1:** Summary Statistics of the Dependent and Independent Variables Used in the Analysis

Variable	*M*	*SD*	Cronbach α
NEET^a^	0.149	0.356	
Education of the mother			
Higher^a^	0.133	0.340	
Secondary^a^	0.343	0.475	
Lower^a^	0.523	0.499	
Education of the father			
Higher^a^	0.134	0.341	
Secondary^a^	0.340	0.474	
Lower^a^	0.526	0.499	
Quality of relationship^b^	3.115	0.548	0.942
Intrusiveness^b^	2.112	0.477	0.840
Support^b^	3.580	0.601	0.666
Autonomy^b^	3.418	0.646	0.736

^a^*n* = 8658. ^b^*n* = 8049.

As can be seen from Table 1, NEETs comprise about 15% of the sample, a slightly lower percentage than that reported by official statistics (21%) due to the fact that, as explained above, we chose to exclude from the NEET category respondents who had not been studying or working for less than six months.

We decided to perform a gender-specific analysis in order to evaluate the different impacts of the explanatory factors on males and females. Indeed, Table 2 shows that for some crucial explanatory variables such as respondents’ Educational level of mothers (only with respect to tertiary education levels), Intrusiveness, and Support, there is a statistically significant difference in means. In particular, the score for the factor “Intrusiveness” is higher among males. Conversely, Support is higher in the females’ subsample.

**Table 2:** Summary Statistics of the Dependent and Independent Variables Used in the Analysis, by Gender

Variable	*M* (Males)	*M* (Females)	Difference
NEET^a^	0.138	0.160	-0.022**
Education of the mother			
Higher^a^	0.143	0.125	0.018*
Secondary^a^	0.333	0.348	-0.015
Lower^a^	0.523	0.526	-0.003
Education of the father			
Higher^a^	0.140	0.130	0.010
Secondary^a^	0.329	0.344	-0.015
Lower^a^	0.530	0.526	0.004
Quality of relationship^b^	3.110	3.116	-0.006
Intrusiveness^b^	2.130	2.096	0.034**
Support^b^	3.556	3.601	-0.045***
Autonomy^b^	3.431	3.408	0.023

^a^*n* = 8658. ^b^*n* = 8049.

*p < .05. **p < .01. ***p < .001.

Results relative to the explanatory variables included in Table 3, in addition to the gender disparity in the percentage of NEETs, suggest the strategy of running two separate models for males and females henceforth.

**Table 3:** Marginal Effects for Predictors of the Condition of NEET

Variables	Males (*n* = 3494)		Females (*n* = 3661)	
	Marginal Effects	*z*	Marginal Effects	*z*
Education of the mother		
Higher	-0.0874***	-3.78	-0.0786**	-3.20
Secondary	-0.0483***	-3.53	-0.0781***	-5.37
Primary or less	0	0	0	0
Education of the father		
Higher	-0.0853***	-3.65	-0.0690**	-2.83
Secondary	-0.0379**	-2.81	-0.0398**	-2.77
Primary or less	0	0	0	0
Quality of relationship	0.0161	1.17	-0.0002	-0.01
Intrusiveness	0.0170	1.44	0.0385**	2.86
Support	-0.0342**	-3.10	-0.0263*	-2.03
Autonomy	-0.0306**	-3.15	-0.0186	-1.87
Pseudo *R*^2^	0.15		0.12	

*p < .05. **p < .01. ***p < .001.

Table 3 provides results for the determinants of the condition of NEET in Italian young adults. The table reports marginal effects for the covariates and *z* statistics. The Marginal Effect of each independent variable measures the average change in the probability of observing a value 1 (the condition of NEET) by an increase of one unit in the explanatory variable. Moreover, we note that we have controlled for respondents’ geographical heterogeneity by introducing macro area dummies in the regression (omitted by the output).

Our results show that parents’ education plays a relevant role in predicting the condition of NEET. In particular, the higher the mother’s education is, the lower the probability that her child will be NEET. More specifically, net of the other covariates, mother’s university education decreases by about 8% the likelihood of being a NEET for both males and females. A secondary education has a smaller impact for males (about 4%) and a larger one for females (more than 7%).

Father’s education also has a significant and monotone effect in reducing the likelihood that his child will be NEET: having a university educated father decreases by over 8% the risk of being a NEET for males and by 7% for females in comparison to lower education (chosen as reference category). As expected, father’s secondary education has a smoothed effect (less than 4% for both genders) compared to that of a university education.

The family indices included in the model also present interesting findings.

Q*uality of relationship* does not have a significant impact on the risk of being NEET while *Intrusiveness* has a significant impact only for women. The higher the degree of parents’ intrusiveness in a young woman’s life, the greater is the likelihood that she will fall into a condition of NEET.

The *Support* index is negatively associated with the NEET condition for both females and males even if the effect is stronger for males (with a marginal effect of -3.42% against an effect of -2.63 for females). Finally, *Autonomy* is negatively related to the NEET condition in the males’ subsample with a marginal effect of -3.06%.

Cragg and Uhler's (1970) *R*^2^ is equal to 0.15 for males’ regression and 0.12 for females’ regression. In this framework we should point out that probit regression does not have an equivalent to the *R*^2^ that is found in OLS regression. Although this statistic ranges between 0 and 1, Cragg and Uhler's (1970) pseudo *R*^2^ does not mean what *R*^2^ means in OLS regression: 0.15 cannot be explained as the proportion of variance of the response variable explained by the predictors. Hence, we suggest caution in considering as totally negative the response of Pseudo *R*^2^. Nevertheless, we acknowledge that Pseudo *R*^2^ values of 0.12 or 0.15 are relatively low. This result is certainly a limitation in our analysis that was caused by the limited set of explanatory variables available in the analysis. More specifically, the NEET condition may not depend only on family indicators and the family of origin’s educational background. The respondents’ occupational condition may have been mainly caused by the macroeconomic context and individual material conditions that are not included in the model in order to avoid reverse causality problems.

## Discussion

The present work aimed to investigate with an exploratory approach a new emerging social phenomenon in industrialized societies. Although there has been some research that has investigated which factors influence NEET status, we found no such studies conducted in the Italian context.

An initial result that emerged from the study is that parents’ educational level has a protective effect on the risk of falling into NEET status for both genders. This finding is coherent with the results of other research (see, for example, Bynner & Parsons, 2002). In particular, Rennison et al. (2005) maintain that parents with low educational levels have less expertise when giving their children advice about educational choices thus guiding their children toward poor and inefficacious choices or toward making no choice at all. Moreover, parents’ educational level can be considered an indicator of socio-economic status (Abramson, Gofin, Habib, Pridan, & Gofin 1982). Education, especially a university degree, requires a large economic investment. This implies that, especially in countries without active labor market policies, young people who cannot count on parents’ help in order to continue their studies tend to leave school prematurely, with a higher risk of being trapped in temporary, low paying jobs that, once concluded, lead to the condition of NEET.

With respect to the relational variables considered, the situation is more complex.

As regards relationship quality, it is worth noting that, in and of itself, this variable does not determine NEET status. This result could be imputed to the fact that relationship quality is a “generic” variable that includes different constructs which, perhaps, compensate for one another. The results that emerged when considering the other variables appear to confirm this hypothesis.

A variable that turned out to be statistically significant for NEET status in both genders is support. In the longitudinal study carried out in the UK by Bynner and Parsons (2002), the authors tested the impact of two variables that we could connect to a form of support (parents reading stories to their children and the interest parents demonstrate toward their children) measured when children were 10 years old and then 6 years later. The results reveal that for males only the first variable has a positive impact while for females only the second one does. Pemberton (2008a) reports on the results of a qualitative study in which most of the NEETs interviewed reported low levels of support from parents, a reason for which these young people had to seek role models outside the family.

On the other hand, intrusiveness influences NEET status positively only for females. When young women do not grow up in a context in which they feel free to explore their environment - a context in which they make some choices supported by their parents yet still feel able to express their own opinions - their ability to assume responsibility for choices, to take charge of their lives in an autonomous manner, and their status as NEETs can be impacted.

In Pemberton’s (2008a) qualitative study cited above, the author finds that many of the NEETs interviewed report that they have overly controlling parents.

In contrast, autonomy has an impact only for males, negatively influencing their status as NEETs. This may suggest that the more young people feel free to build their own identity and are supported in doing so, the more they succeed in developing resilience and in finding resources for not falling into the condition of NEET. On the other hand, the more young people are rendered passive and kept in the family nest, the less they will be able to confront the developmental tasks associated with their phase of the life cycle, and, as a result, could end up in the condition of NEET.

## Conclusions

This study provides important information for the implementation of interventions in the Italian context. In the first place, it emerges that the NEET phenomenon is not only an individual problem; thus, it is not possible to resolve the difficulties connected to it by only taking into consideration NEET young people. Rather, we can consider the NEET phenomenon as being multidimensional.

On a purely individual level, it is necessary to improve education/training and skills by reducing early school dropout rates, in particular. Young people who do not make good use of their years in training or education carry a persistent fragility throughout their working lives. Many studies confirm how important and productive it is to invest well in the younger age brackets (Heckman, 2006).

On the level of *relational* quality, while the best known programs aimed at curbing the phenomenon involve only young people (Chen, 2011), involving families, helping them become more aware of their own functioning, and designing targeted interventions with them in mind could provide valuable help in addressing the NEET phenomenon.

Finally, on a societal level, in order to foster an active attitude in young people, it is important to strengthen the link between education/training and work, which becomes more efficacious the more instructional systems are able to provide students with skills that are directly spendable in the job market and consistent with its evolution. For young people who conclude their studies/training or reach the end of a temporary job contract, employment services are necessary that can create an individual development plan by conducting an assessment of a young person’s skills in relation to the demand constituted by available jobs.

This work has some limitations. First, a longitudinal development of the present research perspective would be fruitful: this would allow for the monitoring of possible changes over the course of different ages. For example, it would be interesting to analyze different “pathways” that young NEETs can undertake (e.g. engaging in even short periods of activity/training, initiating alternative and somehow compensatory activities such as volunteerism, etc.). Moreover, it would allow us to test the model described here by investigating antecedent and consequent variables over time, for example, to better understand whether family relationships influence NEET status or whether, vice versa, it is NEET status that influences family relationships. Third, self-report instruments give us only the respondents’ views and are susceptible to social desirability to various degrees; thus, the use of a multi-method approach would be germane. For example, it would be useful to probe some of the findings that emerged using semi-structured interviews (for example, the quality of relationships with parents). Finally, the values of pseudo *R*^2^ are quite low. It was argued that pseudo *R*^2^ should not be understood as the more common *R*^2^, yet the values are not completely satisfactory. It makes sense, therefore, to hypothesize some additional variables that could be included in the model. Future developments of the present work will seek to overcome these limitations.
